# Comparative Analysis of Fatigue Energy Characteristics of S355J2 Steel Subjected to Multi-Axis Loads

**DOI:** 10.3390/ma13112470

**Published:** 2020-05-28

**Authors:** Cyprian T. Lachowicz, Robert Owsiński

**Affiliations:** Department of Mechanics and Machine Design, Opole University of Technology, ul. Prószkowska 76, 45-758 Opole, Poland; c.lachowicz@po.edu.pl

**Keywords:** energy fatigue model, elasto–plastic strain energy, constitutive models, bending and torsion, kinematic and dynamic loading

## Abstract

In this study, a novel test system for estimating bending and torsion fatigue under selectable kinematic and dynamic loading modes was constructed. Using S355J2 steel specimens, a series of tests were conducted to determine material sensitivity to different load paths and loading modes. The experimental results were supplemented with the results of numerical analyses, on the basis of which the components of strain and stress tensors for subsequent analyses were determined in the entire working part of the specimen. An original method for determining specific strain energy components was used. The experimental results showed the grouping of data according to the mode of loading chosen. This could signify that the selected fatigue models are sensitive to certain loading scenarios. We performed in-depth data analysis and complex numerical simulations, formulating likely explanations for the observed effect.

## 1. Introduction

Non-monotonic alternation of stress states may lead to permanent changes in the structure of materials and is often the cause of the limited functionality of components. The vast majority of machines and engineering structures are subjected to complex and time-varying operational loads [1,2,3,4,5,6]. Generally, the loads can be dynamic, i.e., caused by forces and moments varying in time. They can also be kinematic loads, caused by time-varying displacements and rotations. In both cases, a complex field of strain and stress arises in the structure. Industrial examples, where such loading scenarios are important, include heavy vehicles used for landscaping, such as diggers or crushers. Operation of such equipment requires completing target kinematic displacement against a uncontrollable and unpredictable dynamic reaction from soil or processed material. A reverse case example can be found in many torsionally loaded shafts, where the kinematic response is stochastic, such as large bore drills in the oil and gas industry.

The fatigue properties of construction materials under uniaxial loads are best described in the literature [7,8,9]. However, due to the fact that in reality complex loading states occur in machines and structures, it is more desirable to test the complex state of loading complexity [10,11,12]. The issues that are currently raised in the literature regarding fatigue of materials are the analysis of the correlation between the material microstructure and fatigue life [13,14] as well as issues on the border of fracture mechanics and surface quality [15,16]. Under the influence of non-proportional loadings, the principal stress and strain directions change cyclically [17,18] and, as proven by various studies [17,19], the fatigue life of a given structure can be significantly reduced. A number of studies on a certain group of materials [20] showed a clear impact of material non-proportional hardening sensitivity on the observed fatigue life.

Most experimental studies conducted under multiaxial fatigue load conditions were performed for independent tension: compression and torsion [21,22]. The results of material testing under other loading conditions, for example, for bending with torsion, appear much less frequently in the literature. In most cases, the tests were performed phase-aligned with controlled values of bending and torsional moments amplitudes (dynamic load) [23,24,25,26,27]. Testing of materials subjected to bending and torsional loads, with controlled kinematic load, is much less common; however, some studies [28,29] analyzed essentially aligned kinematic loads causing bending and torsion. Literature sources on material testing in both possible load control variants (dynamic and kinematic) is lacking. In this context, the knowledge of fatigue of materials subjected to phase-dependent kinematic or dynamic loading seems to be underdeveloped, especially given potential significance to many structures and mechanisms. As a part of a research program, material specimens were tested with controlled, with independent kinematic or dynamic loads. A custom-built fatigue testing machine was implemented to facilitate this project. Similar to typical tensile–compression tests with torsion, fatigue tests were performed for both out-of-phase loads and in-phase loads for bending and torsion alone and combinations thereof.

The main objective of this study was to examine the fatigue properties of S355J2 steel using an energetic description. In this work, fatigue tests were conducted on specimens in kinematic and dynamic scenarios with four paths of loadings. The cyclic stress–strain response of the fatigue-tested specimens were analyzed using finite element method (FEM) analysis with the Chaboche model of cyclic plasticity. We determined the effectiveness of the most popular energy models for many variants of cyclic loads based on the original method of determining specific strain energy components to describe fatigue life. The fatigue lives of these specimens were compared between kinematic and dynamic loading test conditions.

## 2. Materials and Methods

### 2.1. Test Specimen

**Figure 1:**
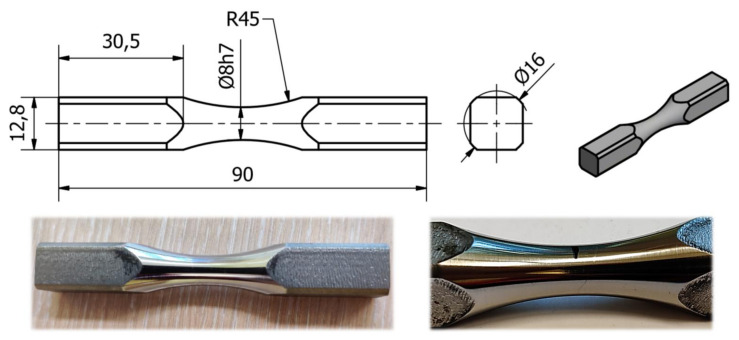
Test specimen. All dimensions are provided in millimeters.

Steel grade S355J2 was supplied for testing in the form of drawn bars with a diameter *ϕ* = 16 mm. The chemical composition of this material was previously published [26], whereas its relevant mechanical properties are listed in Table 1. The test specimen is shown in Figure 1. The chosen shape of specimens ensured that the maximum normal and tangential stresses occurred in the smallest cross-section area, making it the most probable location of fatigue crack initiation.

### 2.2. Fatigue Machine

**Figure 2:**
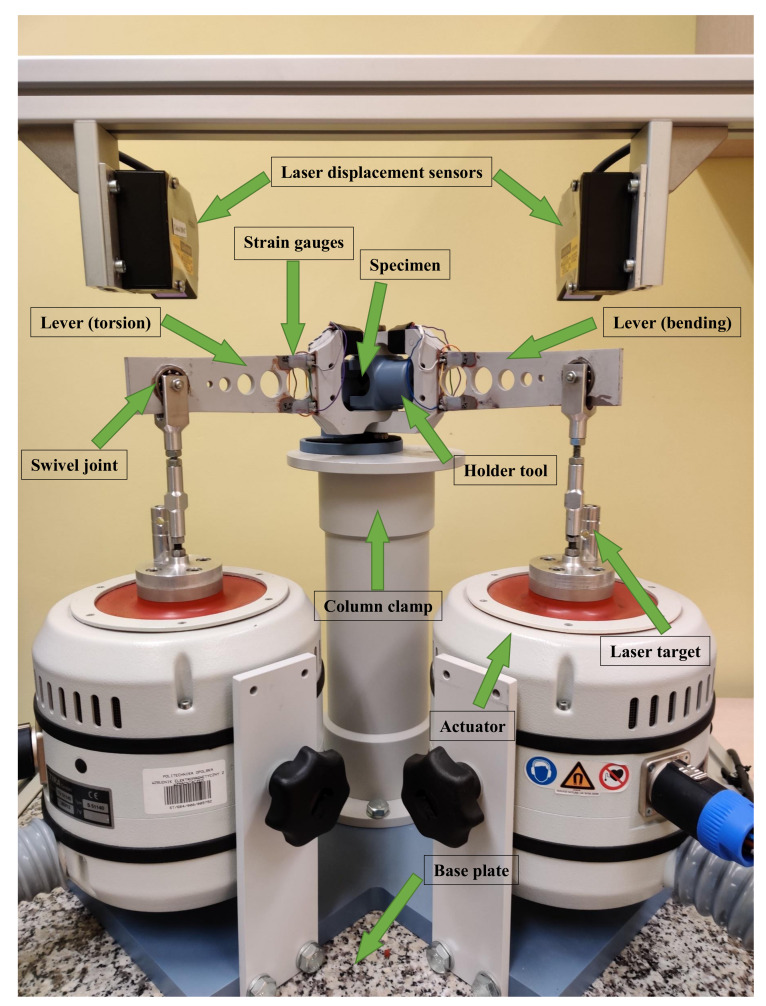
Fatigue testing machine.

The custom-built test stand we used in our research is presented in Figure 2. The fatigue machine rests on a desktop base plate, supported by a frame structure. The test specimen is gripped between a tool holder and a column clamp. Each loading lever (bending and torsional) is connected to the holder tool and through a swivel joint, linked with an actuator that provides excitation.

**Figure 3:**
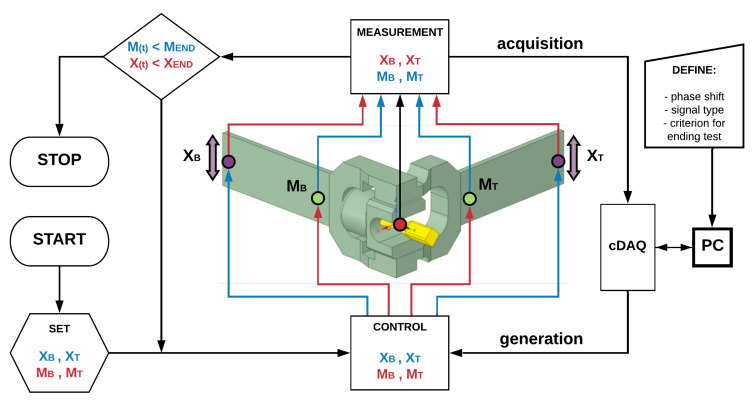
Fatigue machine control and conditioning system.

A schematic diagram of the control and conditioning system used in the experiments is shown in Figure 3. It is based in NI cDAQ hardware (National Instruments, Austin, TX, USA).

A test specimen (Figure 1) was mounted in a tool holder that allows bending and torsional loads to be applied independent of each other. A computerized control system enabled the implementation of any load path and two work modes: kinematic and dynamic. In the former, the loads are delivered as displacements, causing independently controllable torsion $X_{T}$ or bending $X_{B}$, as well as a any combination of both. The maximum permissible range of lever movements was set to ±9 mm. However, experiments were conducted in the range of ±3 mm. Control and acquisition of displacement values were achieved with dedicated laser displacement sensors, monitoring each load lever. In the second dynamic mode of operation, the specimen was loaded dynamically using independently controlled moment of force: torsion $M_{T}$ and bending $M_{B}$. In this case, the permissible range of lever displacement was also limited to ±9 mm. The values of bending and torsional moments specified in the experiment were within the range of ±28 Nm. The control and acquisition of the moment values were carried out using appropriately calibrated strain gauges located on the load levers. The specimen neck (its smallest diameter, see Figure 1) caused a concentration of the maximum elastic and plastic strains. The average components of the $\varepsilon_{i,j}$ strain tensor at a selected measuring point can be measured using resistance strain gauges. However, this required sticking a flat strain gauge on the saddle surface of the specimens’ neck. Even with relatively small strain gauges, their deformation and averaged measured deformation values must be considered. We decided to calculate the values of strain tensor components by indirect means, as described in detail below. The control and measurement system also acquired strain value data at the specimen neck. These data were used for preliminary calibration to determine the relationship between lever displacements and strain measured at the neck, which aided in later numerical modelling work.

### 2.3. Research Methodology

**Figure 4:**
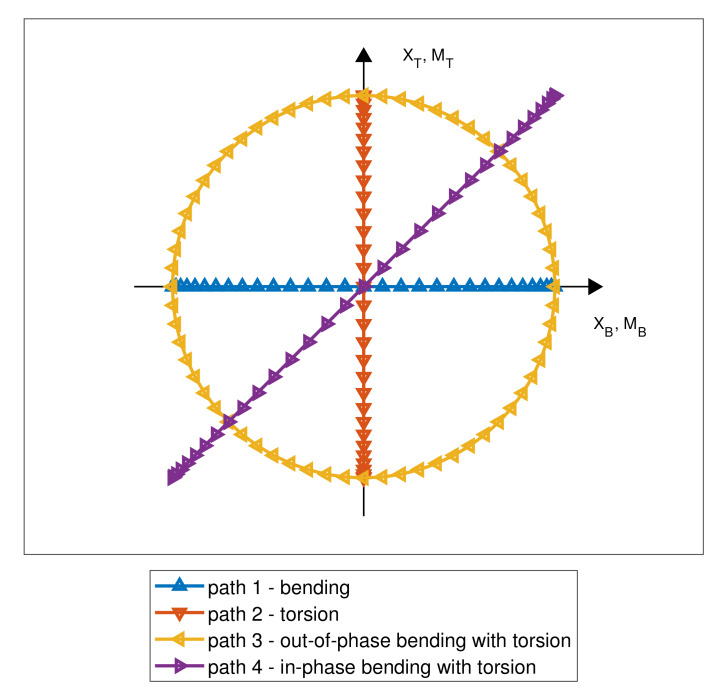
Loading paths.

In the conducted experiments, we assumed that the respective ratios of bending and torsion displacements or moments of force amounted to unity. All loading paths used in experiments were fully reversed, as shown in Figure 4, as follows: path I, bending; path II, torsion; path III, proportional in-phase bending with torsion; and path IV, out-of-phase bending with torsion. As the experimental tests were conducted using kinematic and dynamic modes, two different criteria for specimen failure were adopted. For kinematic loads, the number of cycles to specimen failure was defined as the durability corresponding to a 20% change in the measured bending or torsional moment. For dynamic loads, the fatigue life was defined as number of cycles until a 20% change in the displacement of the bending or torsional lever was achieved. All tests were conducted at an excitation frequency of 20 Hz.

### 2.4. Experimental Results

Table 2 summarizes the obtained values of fatigue life for specimens tested under controlled dynamic or kinematic loads. Specimens marked as *H* were subjected to kinematic loading, whereas specimens marked as *P* were subjected to dynamic loading.

### 2.5. Numerical Determination of Strain and Stress State Components

For construction materials in an elastic state, stress can be easily calculated directly on the basis of strains (or vice versa, strains on the basis of stress). In this case, the generalized Hooke law can be used. However, this approach ceases to be adequate as soon as the first plastic strain appears. In this case, the relationship between the strain and stress is strictly dependent on the load trajectory. Then, a constitutive model of cyclic plasticity must be used. Constitutive models, despite their diversity in terms of scope of applications and phenomena they embrace, always consist of three basic components.

- The plasticity condition in which the von Mises equation is the most commonly used
  (1) $$f\left(\underline{\underline{s}},\underline{\underline{\alpha }},\sigma_{y}\right)=\frac{3}{2}\left(\underline{\underline{s}}-\underline{\underline{\alpha }}\right):\left(\underline{\underline{s}}-\underline{\underline{\alpha }}\right)-\sigma_{y}^{2}=0,$$
  where $\underline{\underline{s}}$ is the stress deviator, $\underline{\underline{\alpha }}$ is the back stress tensor, $\sigma_{y}$ is the radius of the plasticity surface (the limit of cyclic plasticity in uniaxial tensile test), the mark “:” represents the scalar product of two tensors, and $\underline{\underline{\;}}$ denotes a tensor.
- Associated flow rule, which allows the determination of the increase in plastic strain, which, according to Drucker’s postulate [30], is normal to the plasticity surface
  (2) $$d{\underline{\underline{\varepsilon }}}^{p}=\frac{1}{H}\left(d\underline{\underline{s}}:\underline{\underline{n}}\right)\underline{\underline{n}},$$
  where $d\underline{\underline{s}}$ is the stress deviator increase, $\underline{\underline{n}}$ is the normal vector for plasticity surfaces $\underline{\underline{n}}=\sqrt{\frac{3}{2}}\frac{\left(\underline{\underline{s}}-\underline{\underline{\alpha }}\right)}{\sigma_{y}}$, and *H* is the plasticity module.
- The hardening rule. In most cases, to calculate fatigue, the kinematic hardening rule is used, allowing the position of plasticity surface $\underline{\underline{\alpha }}$ to be determined after each increment in the stress deviator $\underline{\underline{s}}$. For example, for the non-linear Chaboche model [30,31], the hardening rule takes the following form:
  (3) $$d\underline{\underline{\alpha }}=\sum_{i=1}^{3}\left(\frac{2}{3}C_{i}d{\underline{\underline{\varepsilon }}}^{p}-D_{i}{\left(\frac{2}{3}d{\underline{\underline{\varepsilon }}}^{p}:d{\underline{\underline{\varepsilon }}}^{p}\right)}^{\frac{1}{2}}{\underline{\underline{\alpha }}}_{i}\right),$$
  where $C_{i}$ and $D_{i}$ (Table 1) are the parameters of the cyclic strain curve function [32,33]
  (4) $$\sigma_{a}=\sigma_{y}+\sum_{i=1}^{3}\frac{C_{i}}{D_{i}}\tanh D_{i}\varepsilon_{a}^{p}.$$

To determine the components of the strain and stress state in specimens prior to experiments, a finite element method simulation was conducted on a numerical model of the test stand, produced in ANSYS 2019 R1 software (Ansys, Canonsburg, PA, USA). This allowed the computation of the field of stress $\underline{\underline{\sigma }}\left(t,x,y,z\right)$ and strain $\underline{\underline{\varepsilon }}\left(t,x,y,z\right)$ tensors in the specimen when subjected to loadings in form of moments or displacements.

**Figure 5:**
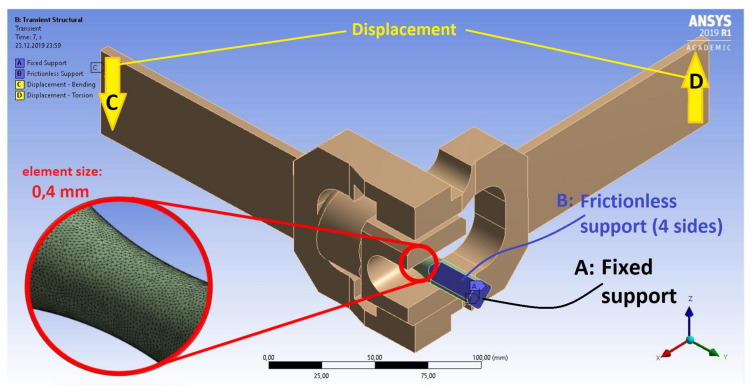
Numerical model of clamping/loading system of the fatigue machine.

The numerical model of the fatigue machine consisted of two levers, a holder tool, and a test specimen (Figure 5). These virtual components were supported and loaded to reflect the kinematics of fatigue machine operation. Although some minor geometrical features of the design were simplified, clamping and loading remained unaffected compared to the actual test stand. A detailed view in Figure 5 depicts the FEM model mesh structure (Table 3) for the virtual specimen, which consisted of 245,561 finite elements distributed over the neck region of the specimen. The finite element mesh was optimized using standard ANSYS procedures. The FEM model uses a diverse finite element mesh that was optimized for the given calculation model.

**Figure 6:**
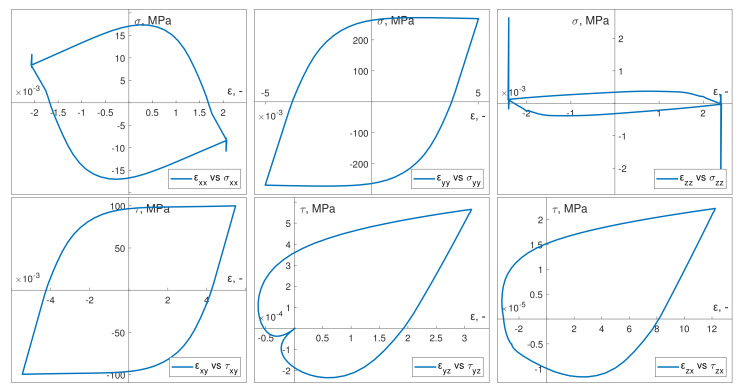
Numerical obtained hysteresis loops for in-phase kinematic load.

**Figure 7:**
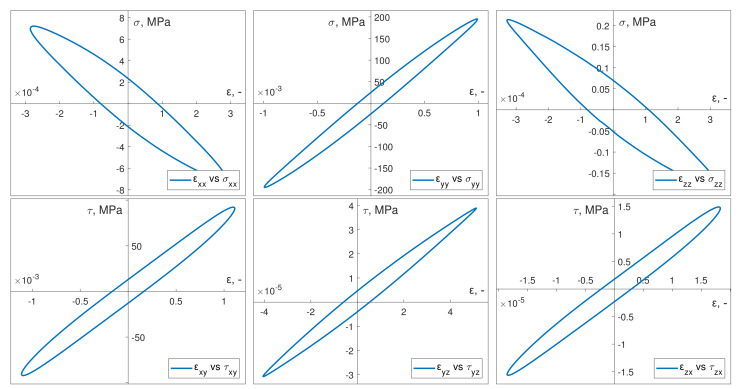
Numerically calculated hysteresis loops for out-of-phase dynamic load.

The cyclic properties and other necessary material data required to introduce the Chaboche constitutive model were determined on the basis of the cyclic strain curves of the S355J2 steel specimens. The loading conditions of the specimen, implemented in experiments under kinematic or dynamic loading, ensured that only normal strains $\varepsilon_{yy}$ and shear strains $\varepsilon_{xy}$ (and their corresponding stress values) reached significant values (Figure 6 and Figure 7). Other strain and stress values were significantly smaller.

As the chosen shape of the specimen (Figure 1) guided the fatigue fracture location (within the neck region), further analysis only considered the states of the strain and stress in the selected, most stressed point on the surface of the specimen, as indicated by numerical simulations.

### 2.6. Energetic Description of the Experimental Results

The comparison between the fatigue life of specimens tested with two different load modes (kinematic and dynamic) required the selection of the correct fatigue parameter. Among many models found in the literature [32,34,35,36,37], five common energetic models were used:

- Smith–Watson–Topper model

The Smith–Watson–Topper (SWT) $\Delta W^{SWT}$ parameter [38] is based on a product of maximum stress $\sigma_{n,max}$ and principal strain range $\Delta \varepsilon_{1}$ located on the principal strain range plane

(5) $$\Delta W^{SWT}=\sigma_{n,max}\frac{\Delta \varepsilon_{1}}{2}.$$

- Chu model

Chu [39] proposed a fatigue parameter using tensile work $\sigma_{n,max}\frac{\Delta \varepsilon }{2}$ complemented by torsion work $\tau_{n,max}\frac{\Delta \gamma }{2}$:

(6) $$\Delta W^{*}=max\left(\tau_{n,max}\frac{\Delta \gamma }{2}+\sigma_{n,max}\frac{\Delta \varepsilon }{2}\right).$$

- Liu model

For multi-axial loads, Liu [40] proposed a model adopting two different basic modes of fatigue damage. The first is caused by normal stresses, characterized by virtual energy $\Delta W^{\left(I\right)}$. The second mode is produced by shear stress, characterized by virtual energy $\Delta W^{\left(II\right)}$. Fracture is expected on a plane where the value of the virtual energy reaches its maximum. The calculation of the $\Delta W^{\left(I\right)}$ value is preceded by a search for the plane on which the work on the normal strain is the highest. Subsequently, work on shear strain is calculated.

(7) $$\Delta W^{\left(I\right)}={\left(\Delta \sigma \Delta \varepsilon \right)}_{max}+\left(\Delta \tau \Delta \gamma \right).$$

Virtual work is calculated similarly $\Delta W^{\left(II\right)}$. In this case, the value of the work on the shear strain is considered first:

(8) $$\Delta W^{\left(II\right)}=\left(\Delta \sigma \Delta \varepsilon \right)+{\left(\Delta \tau \Delta \gamma \right)}_{max}.$$

- Glinka model

Glinka et al. [41] proposed a fatigue parameter calculated as the sum of normal and tangential stress work on corresponding strains, determined by the plane of maximum shear strains:

(9) $$W^{*}=\frac{\Delta \gamma_{12}}{2}\frac{\Delta \sigma_{12}}{2}+\frac{\Delta \varepsilon_{22}}{2}\frac{\Delta \sigma_{22}}{2}.$$

The authors, aiming to include the impact of mean stresses in the parameter in Equation (9), proposed modifications in [42]:

(10) $$W^{*}=\frac{\Delta \gamma }{2}\frac{\Delta \tau }{2}\left[\frac{\sigma_{f}^{{}^{\prime }}}{\sigma_{f}^{{}^{\prime }}-\sigma_{n,max}}+\frac{\tau_{f}^{{}^{\prime }}}{\tau_{f}^{{}^{\prime }}-\tau_{n,max}}\right],$$

where $\sigma_{f}^{{}^{\prime }},\tau_{f}^{{}^{\prime }}$ are the fatigue strength coefficients for tensile compression and torsion, respectively.

- Ellyin model

The Ellyin model [43,44] proposes both plastic strain energy $\Delta W^{p}$ and positive elastic strain energy $\Delta W^{+}$:

(11) $$\Delta W=\Delta W^{p}+\Delta W^{+}.$$

For selected cases of proportional and non-proportional loads, the energy is calculated using the following formula:

(12) $$\Delta W=\int_{t}^{t+T}\sigma_{ij}d\varepsilon_{ij}^{p}+\int_{t}^{t+T}H\left(\sigma_{i}\right)H\left(d\varepsilon_{i}^{e}\right)\sigma_{i}d\varepsilon_{i}^{e},$$

where $\sigma_{ij}$ and $\varepsilon_{ij}^{p}$ are a stress tensor and plastic strain tensor, respectively; $\sigma_{i}$ and $\varepsilon_{i}^{e}$ are the stress and elastic parts of the principal strains, respectively; and H(x) is the Heaviside function.

### 2.7. Calculation and Identification of Specific Strain Energy Components

When applying the Ellyin model, which refers directly to the elasto-plastic strain energy density, it was necessary to calculate it properly using the strain and stress tensor time curves determined previously through numerical simulations.

**Figure 8:**
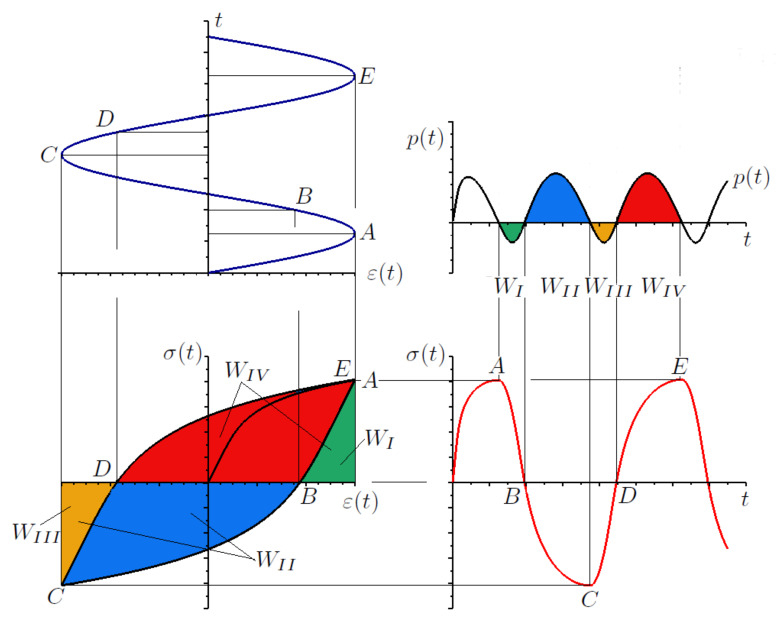
Method of calculating the elastic–plastic strain energy density by integrating the time course of instantaneous power.

Previous studies [19,45] presented an application case for the methodology described in [46]. Through the proposed instantaneous power, defined as:

(13) $$p=\frac{\delta W}{dt}\equiv \sigma_{ij}\frac{d\varepsilon_{ij}}{dt}=\sigma_{ij}{\dot{\varepsilon }}_{ij},$$

a consecutive time sequence of instantaneous steady states can be presented with the following equation:

(14) $$p\left(t\right)=\sigma_{ij}\left(t\right){\dot{\varepsilon }}_{ij}\left(t\right).$$

where δW is an increment of work of the internal forces on the infinitesimal elongation/shortening increments. Figure 8 presents the method of calculating the strain energy density for cyclic elasto-plastic loadings. The computational methodology is illustrated on an example of uniaxial tension-compression; however, it is analogous to bending and torsion with respect to the change in the selected strain or stress tensor values. The specified extent of loading variations consists of two ranges: A–C, which is the primary range, and C–E, which is the complementary range. Within the first, another distinction can be made between the tension relief (A–B) and compression (B–C) ranges. Similar to the primary range, the complementary range also comprises both a compression relief range (C–D) and a tension range (D–E). The area cut off by the power time course, as a result of the work of internal forces during a relief phase, is represented by $W_{I}$. This is followed by the $W_{II}$ corresponding to the strain energy density related to the work of external forces throughout the compression phase and the $W_{III}$ representing the work of internal forces during the relief after compression phase. Finally, $W_{IV}$ denotes the strain energy density related to the work of the external forces while the material was undergoing the tension phase. Notably, the sum of the areas that ware cut off by of the power time course is not equal for regions located above and below the time axis. By adding $W_{II}$ and $W_{IV}$ and subtracting the result by the sum of $W_{I}$ and $W_{III}$, the plastic strain energy density can be calculated for the specified pair of ranges (hysteresis loop).

(15) $$\left(W_{II}+W_{IV}\right)-\left(W_{I}+W_{III}\right)=W^{p}.$$

Figure 8 presents the location of areas $W_{I}$, $W_{II}$, $W_{III}$, and $W_{IV}$ in relation to the hysteresis loop formed by applied loading.

This method of identification and calculation of the total strain energy density components [46], as described above, can be applied to the results of the FEM simulations.

**Figure 9:**
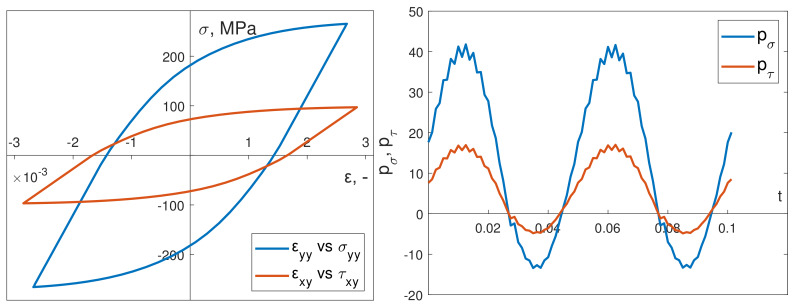
Stabilized calculated hysteresis loops for kinematic in-phase load and calculated instantaneous power (bending) $p_{\sigma }$ and torsion $p_{\tau }$.

**Figure 10:**
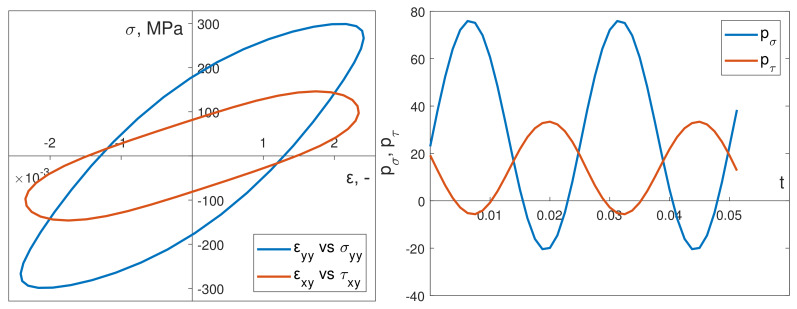
Stabilized calculated hysteresis loops for kinematic out-of-phase load and calculated instantaneous power (bending) $p_{\sigma }$ and torsion $p_{\tau }$.

Figure 9 depicts an example of a stabilized hysteresis loop and instantaneous power time courses for bending $p_{\sigma }$ and torsion $p_{\tau }$ in the numerical simulations. These were conducted in-phase in kinematic loading mode. Figure 10 shows the corresponding numerical data results from out-of-phase kinematic load simulations.

**Figure 11:**
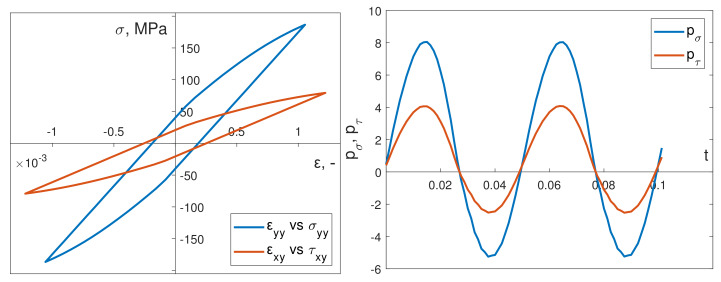
Stabilized calculated hysteresis loops for dynamic in-phase load and calculated instantaneous power (bending) $p_{\sigma }$ and torsion $p_{\tau }$.

**Figure 12:**
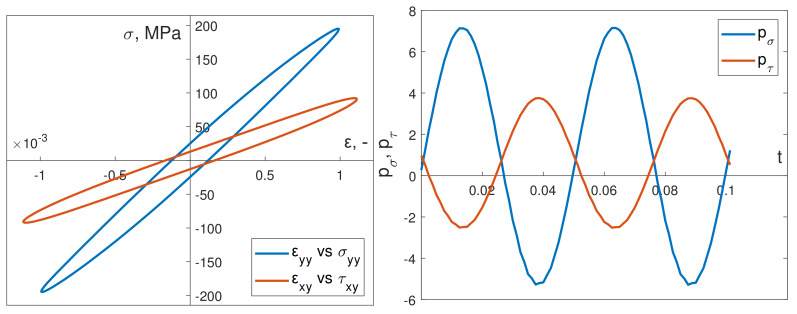
Stabilized calculated hysteresis loops for dynamic out-of-phase load and calculated instantaneous power (bending) $p_{\sigma }$ and torsion $p_{\tau }$.

Figure 11 illustrates an example of the instantaneous power time course for bending $p_{\sigma }$ and torsion $p_{\tau }$, captured in simulations, under dynamic in-phase loading. In addition, Figure 12 presents the corresponding results for dynamic out-of-phase loading simulations.

## 3. Results and Discussion

Table 4 and Table 5 summarize the results of the numerical calculations of the elastic–plastic strain energy Equation (11) and the fatigue energy parameters from Equations (5) to (10). ASTM E739 standard recommendations were used to assess suitability of the selected fatigue parameter for describing the performed fatigue tests. Following ASTM standard guidelines [47], a linear regression model was adopted (presented in a double logarithmic system) to assess the suitability of the selected fatigue parameter

(16) $$\mathrm{lg}N_{f}=A+B\mathrm{lg}W,$$

where $N_{f}$ is durability expressed in cycles, *W* is the value of the strain energy or fatigue energy parameter, and *A* and *B* are parameters calculated for this regression model. Table 6 summarizes the calculated values of the model parameters in Equation (16) and the values of the correlation coefficient $\varrho_{xy}$.

Values of $\varrho_{xy}$ differ depending on the mode used for specimens loading. For kinematic loads, the values are similar and not less than 0.9, with the exception of the Glinka model, where $\varrho_{xy}=0.62$. For dynamic loads, the values of $\varrho_{xy}$ are significantly lower, in the range of 0.47 to 0.8.

All considered energy models achieved good correlation with experimental data regardless of loading path, but only within each loading mode used in the experiments (either kinematic or dynamic).

**Figure 13:**
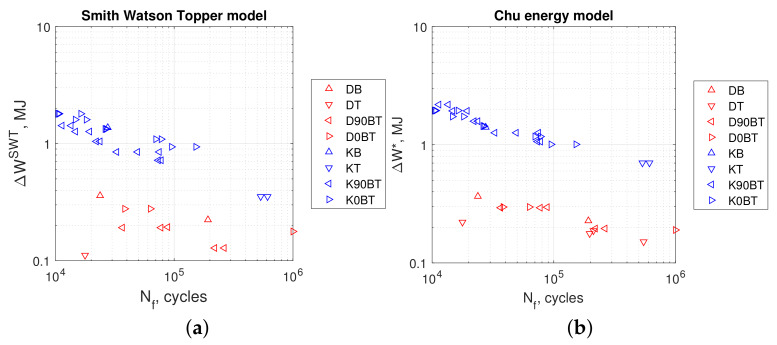
(**a**) Smith Watson Topper model in Equation (5), (**b**) Chu model in Equation (6). Tests at kinematic load: K90BT, out of phase; K0BT, in phase; KB, bending; KT, torsion. Dynamic loads: D90BT, out of phase; D0BT, in phase; DB, bending; DT, torsion.

**Figure 14:**
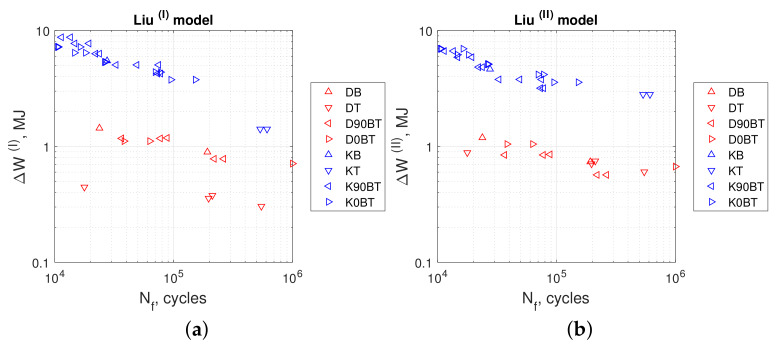
(**a**) Liu ${}^{\left(I\right)}$ model Equation (7), (**b**) Liu ${}^{\left(II\right)}$ model Equation (8). Tests at kinematic load: K90BT, out of phase; K0BT, in phase; KB, bending; KT, torsion. Dynamic loads: D90BT, out of phase; D0BT, in phase; DB, bending; DT, torsion.

**Figure 15:**
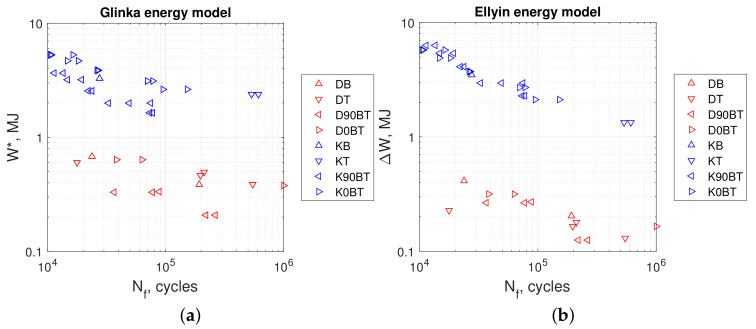
(**a**) Glinka parameter in Equation (9), (**b**) Ellyin energy parameter in Equation (11). Tests at kinematic load: K90BT, out of phase; K0BT, in phase; KB, bending; KT, torsion. Dynamic loads: D90BT, out of phase; D0BT, in phase; DB, bending; DT, torsion.

Figure 13a,b, Figure 14a,b, and Figure 15a,b show the calculated values of energy parameters (Equations (5)–(9)) and strain energy (Equation (11)) as a function of fatigue life. For all energy models, we noticed that the values obtained in kinematic loading mode were clearly separated from the results acquired in dynamic loading mode. Both groups formed distinct scatter bands in charts, which seemed to be broadly parallel to each other.

All models described above use the work of stress on strains. However, only the Ellyin model considers the hysteresis loop directly in the calculation of strain energy. The SWT, Liu, Chu, and Glinka models propose the use of certain values describing the state of stress and strain that are relatively easy to calculate. However, according to Liu [40], their relationship with the plastic strain energy is abstract. It is especially obvious for non-proportional loads, for which these models were not originally intended for use.

Evaluating the results presented in Table 5 and Table 6, we observed that the values of Liu’s energy parameters were always greater than strain energy calculated from Ellyin’s models. This was noticeable for both loading modes: kinematic and dynamic. For the former, the SWT, Chu, and Glinka energy parameter values were smaller than strain energy calculated from Ellyin’s equations. For the latter mode, a different situation occurred. In this case, both SWT and Chu’s parameters return values close to Ellyin’s, whereas results calculated according to Glinka are noticeably larger.

Comparing the durability under different load methods (kinematic and dynamic), we found that the value of elastic–plastic strain energy or any energy parameter can be significantly different for the same achieved fatigue life.

**Figure 16:**
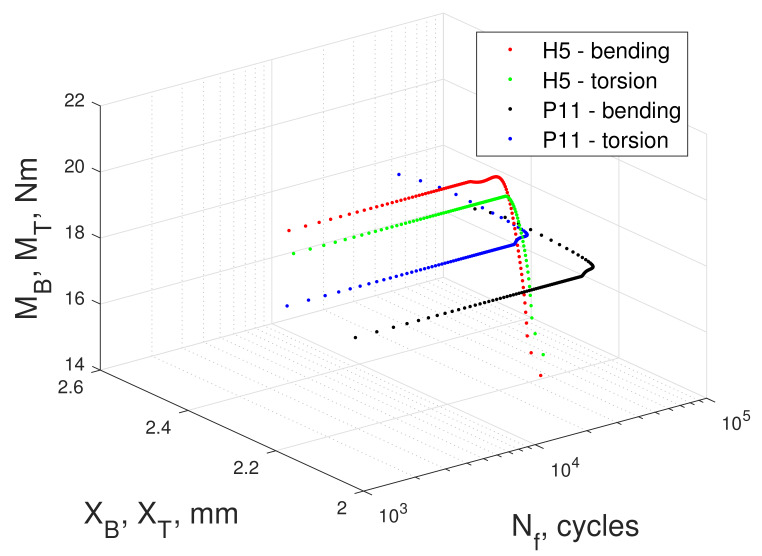
Change in recorded values of bending and torsional moments of specimen No. H5 during the entire test period under kinematic load (red and green) and change in the value of the machine lever displacement for dynamic load for specimen no. P11, black and blue.

**Figure 17:**
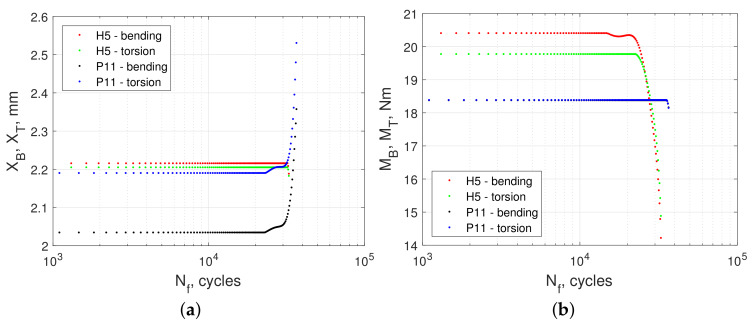
(**a**) Change in the fatigue testing machine lever displacement throughout the entire duration of the specimen test. Red and green correspond to the specimen subjected to kinematic loading, whereas black and blue correspond to the fatigue testing machine lever displacement for the specimen subjected to dynamic loading, (**b**) The moments load the specimen throughout the entire test period. Red and green correspond to the specimen subjected to kinematic loading, whereas black and blue correspond to the specimen subjected to dynamic loading.

Searching for the underlying reasons for the difference in strain energy between kinematic and dynamic loading modes, we directly analyzed the time courses of the moment of force and displacements of bending and torsion levers. Figure 16 and Figure 17 depict the total work imputed into the excitation system: levers, holder, and specimen. The rigid design of the test stand ensured that only an insignificant amount of that energy was spent outside of the specimen, dissipated through inter-component clearances, fastened connections, bearings, etc.

Figure 16 presents the curves $f_{kin,dyn}\left(X_{B,T},M_{B,T},N_{f}\right)$ showing the relationship between the life $N_{f}$ of the specimen, displacement $X_{B,T}$, and the moment of force $M_{B,T}$ recorded at the levers. Two example specimens were selected, H5 (kinematic mode) and P11 (dynamic mode), which exhibited similar fatigue life. The curve marked in red $f_{kin}\left(X_{B},M_{B},N_{f}\right)$ and the curve marked in green $f_{kin}\left(X_{T},M_{T},N_{f}\right)$ correspond to bending and torsion lever in kinematic mode, respectively. This chart also depicts the dynamic mode results, where the black curve $f_{dyn}\left(X_{B,T},M_{B,T},N_{f}\right)$ indicates bending and the blue curve $f_{dyn}\left(X_{T},M_{T},N_{f}\right)$ denotes torsion.

Figure 17b illustrates that the tests conducted under kinematic load (lines in red and green) returned a near constant value of amplitudes of moments of force for approximately 5/6 of the the fatigue test duration, only to start dropping near the end of the test. The amplitude of displacements (Figure 17a) did not change throughout the experiment in this mode of loading (kinematic). Throughout the first 5/6 of the test, instantaneous strain energy was constant; however, as the moment of force decreased in the final stage of the experiment, so did the strain energy.

A similar effect was observed for tests under dynamic loading. However, in this case, the observed effects were different. Figure 17a depicts an increase in the amplitude of both lever displacements (black and blue curves) at a constant value of moment loads (Figure 17b). Notably, in both figures, the black lines correspond to the bending and blue to the torsion. Here, at the conclusion of the tests, the amplitude of displacements increased. Since the values of the moment of force amplitude did not change (Figure 17b), the value of the strain energy must increase when dynamic load mode is used.

Among the considered energy fatigue parameters, none consider the temporal transiency of strain tensor and the corresponding stress tensor. As a consequence, the values of strain energy (stress work on strains) were calculated with these models for a given time instance. Thus, the results ignore the changes in the values of the strain and stress tensor components during the fatigue process, affecting the strain energy or any energy parameter calculations.

The numerical constitutive cyclic-plasticity model (Chaboche) used in calculations does not consider an important transiency in material properties caused by damage accumulation or crack initiation, propagation, etc. As a result, the obtained strain and stress tensor values are likely impacted.

A second limitation of the considered energy fatigue models is related to their spatial sensitivity (local effect). This is understood as the need to calculate strain energy only at the most stressed point in the smallest cross-section of the specimen. This, in addition to sampling at an arbitrarily determined time instance, cannot provide complete information on the distribution of strain energy in a representative volume of the specimen. This volume can be considered to be subjected to plastic strains.

**Figure 18:**
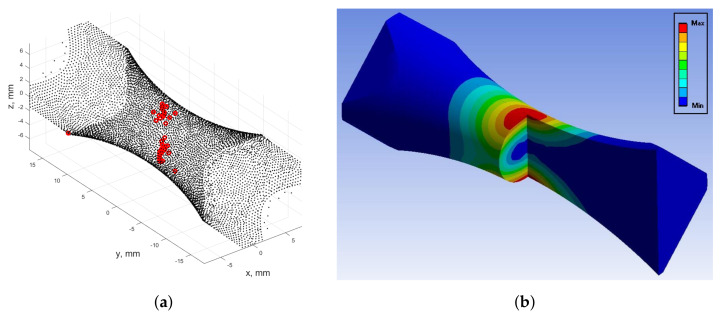
(**a**) Distribution of the most stressed mesh nodes calculated according to the von Mises criterion for in-phase kinematic loading; (**b**) Area of occurrence of plastic strains finite element method (FEM) calculated for the Chaboche cyclic plasticity model for in-phase kinematic loading.

Figure 18a depict the distribution of mesh nodes at the specimen neck during in-phase kinematic loading. The numerical model expressed the highest levels of stress at the two opposing node regions located on both sides of the neck (marked in red). The plastic stain distribution for this loading case is illustrated in Figure 18b.

**Figure 19:**
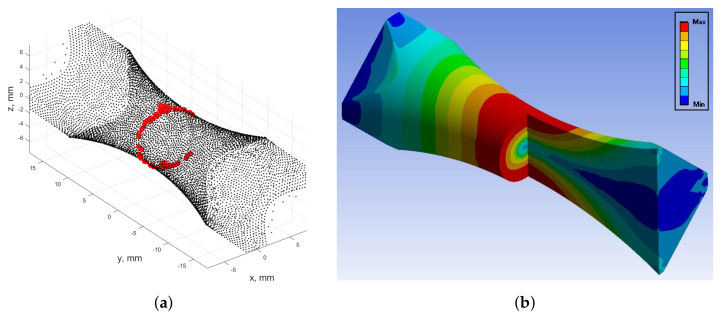
(**a**) Distribution of the most stressed mesh nodes calculated according to the von Mises criterion for in-phase kinematic loading; (**b**) Area of occurrence of plastic strains FEM calculated for the Chaboche cyclic plasticity model for out-of-phase kinematic loading.

In comparison, Figure 19a demonstrates the corresponding distribution of mesh nodes during out-of-phase kinematic loading. Here, the highest stress concentration at the nodes form a ring encircling the narrowest cross-section of the neck. This region corresponds directly to the plastic strain distribution region, as presented in Figure 19b. Similar maximum stress patterns and plastic strain regions were observed for dynamically loaded numerical simulations.

The numerically obtained values of strain energy and energy parameters were expected to be correctly estimated by a single regression model, regardless of loading path and irrespective of loading mode (dynamic or kinematic). Only the former of these assumptions was fulfilled, suggesting that the evaluated common fatigue models might be not entirely applicable in every load case.

## 4. Conclusions

- We conducted experimental research of the bending and torsional properties of S355J2 steel under two different modes of load application, which showed the significant limitations of some popular energy parameters used to determine fatigue durability.
- Despite similarities observed in the fatigue life of specimens tested under kinematic and dynamic modes of load application, neither energy parameters nor strain energy calculations allowed for the common regression model to be implemented for all results. Kinematic and dynamic data required separate regression models.
- The energy parameters published in the literature were in good agreement with data obtained from different loading paths, but only within specific groups of results obtained from either the kinematic or dynamic mode of loading.
- Among the possible reasons for such behavior, we identified the three most likely causes:
  1. The constitutive model of cyclic plasticity (Chaboche) influences the numerically obtained values of strain and stress tensors. The used model assumes that these values are stable and do not change in time, in contrast to actual fatigue damage. This can significantly influence the energy parameter and strain energy values.
  2. The described popular fatigue models consider only a singular arbitrary chosen time instance in fatigue life to compute either the energy parameter or strain energy. This results omit the time dependency in strain energy.
  3. The spatial sensitivity (local effect) of fatigue parameters proposed by many authors is a limitation affect the accuracy of the results. This is because when calculating many energy parameters as a single parameter, only the most stressed location is considered. As a consequence, certain parts of plastic strain energy deposited in the specimen are omitted.
- The custom-designed and built fatigue test stand will enable further investigations into bending and torsion fatigue behavior of materials under many load paths, including random loads and both kinematic and dynamic load application mode.
- As energy models do not consider the change in energy over time associated with the initiation and development of fatigue damage, the variability of energy parameters must be considered during the fatigue test and an algorithm must be developed for estimating energy values over a specific section, area, or volume depending on the analyzed load variant. This will be our future research objective.
- Future work will consider the sensitivity of structural materials to non-proportional hardening under these two loading modes.

## Figures and Tables

**Table 1:** Mechanical properties of S355J2 steel.

$\sigma_{y}$	$\sigma_{u}$	*E*	ν	$K^{{}^{\prime }}$	$n^{{}^{\prime }}$	$C_{1}$	$C_{2}$	$C_{3}$	$D_{1}$	$D_{2}$	$D_{3}$
MPa	MPa	GPa	–	MPa	–	MPa	MPa	MPa	–	–	–
155	535	213	0.29	1325	0.204	309,760	91,832	26,515	4971	930	0

**Table 2:** Experimental test results for S355J2 steel specimens.

No.	$X_{B}$	$X_{T}$	$M_{B}$	$M_{T}$	Loading Path	$N_{f}$
	**mm**	**mm**	**Nm**	**Nm**	**(See Figure 4)**	**Cycles**
**Kinematic Load**						
H1, H2	3	3			3	13,540, 11,360
H3, H4	2.5	2.5			3	22,100, 23,760
H5, H6, H7	2.2	2.2			3	32,780, 74,800, 49,100
H8, H9	2.8	2.8			3	14,760, 19,400
H10, H11	2	2			3	77,720, 73,440
H12, H12, H13	3	3			4	16,380, 10,720, 10,500
H15, H16	2.8	2.8			4	14700, 18,180
H17, H18	2.5	2.5			4	26,200, 26,740
H19, H20	2.2	2.2			4	77,840, 70,080
H21, H22	2	2			4	152,380, 95,040
H26	3	0			1	27,580
H28, H29	0	3			2	535,540, 611,720
P2			20	0	1	192,520
P4			25.64	0	1	23,780
P5			0	24.59	2	197,190
P6			0	25.27	2	211,900
P7			0	27.38	2	17,760
P8			0	22.74	2	547,680
P9, P10, P11			18.36	18.36	3	77,320, 88,240, 36,600
P17, P18			14.84	14.84	3	219,500, 263,720
P40, P41			18.6	18.6	4	38,360, 63,100
P46			14.79	14.79	4	1,002,160

**Table 3:** Analysis detalies.

Quantity		Total	Neck Region
Elements		116,142	111,883
Nodes		260,223	245,561
Elements types		WED15, TET10 (>95%)	
Loading cycles		7 (3 increasing)	
Substeps		80/cycle (560 total)	

**Table 4:** The elastic–plastic strain energy per cycle.

No.	Bending		Torsion				
	$\Delta W_{\mathrm{yy}}^{p}$	$\Delta W_{\mathrm{yy}}^{+}$	$\Delta W_{\mathrm{xy}}^{p}$	$\Delta W_{\mathrm{xy}}^{+}$	$\sum \Delta W^{p}$	$\sum \Delta W^{+}$	ΔW
	**MJ**	**MJ**	**MJ**	**MJ**	**MJ**	**MJ**	**MJ**
**Kinematic Load**							
H1,H2	4.213	0.048	2.048	0.004	6.261	0.053	6.314
H3,H4	2.693	0.072	1.330	0.011	4.024	0.084	4.108
H5,H6,H7	1.901	0.09	0.951	0.017	2.852	0.107	2.96
H8, H9	3.581	0.059	1.748	0.006	5.329	0.065	5.395
H10, H11	1.442	0.098	0.728	0.021	2.17	0.119	2.289
H12, H13, H14	3.935	0.145	1.623	0.052	5.558	0.197	5.755
H15, H16	3.323	0.148	1.376	0.052	4.699	0.200	4.9
H17, H18	2.484	0.151	1.038	0.052	3.523	0.203	3.727
H19, H20	1.758	0.1512	0.745	0.0522	2.503	0.2034	2.706
H21, H22	1.344	0.147	0.575	0.051	1.919	0.199	2.118
H26	3.269	0.216	0	0	3.269	0.216	3.485
H28, H29	0	0	1.176	0.161	1.176	0.161	1.337
**Dynamic Load**							
P2	0.111	0.091	0	0	0.111	0.091	0.203
P4	0.282	0.130	0	0	0.282	0.130	0.412
P5	0	0	0.092	0.072	0.092	0.072	0.165
P6	0	0	0.103	0.075	0.103	0.075	0.179
P7	0	0	0.141	0.086	0.141	0.086	0.227
P8	0	0	0.066	0.064	0.066	0.064	0.130
P9, P10, P11	0.094	0.075	0.060	0.035	0.155	0.110	0.265
P17, P18	0.025	0.058	0.010	0.030	0.035	0.089	0.125
P40, P41	0.131	0.075	0.074	0.035	0.205	0.110	0.316
P46	0.055	0.052	0.031	0.025	0.086	0.078	0.165

**Table 5:** Values of fatigue energy parameters.

No.	$N_{f}$		SWT	Liu ${}^{\left(I\right)}$	Liu ${}^{\left(II\right)}$	Chu	Glinka	Ellyin
	**Cycles**		**MJ**					
**Kinematic Load**								
H1,H2	13,540, 11,360		1.426	8.750	6.660	2.188	3.658	6.314
H3,H4	22,100, 23,760		1.045	6.315	4.833	1.582	2.554	4.108
H5,H6,H7	32,780, 74,800, 49,100		0.849	5.053	3.785	1.263	1.996	2.96
H8,H9	14,760, 19,400		1.269	7.739	5.884	1.935	3.203	5.395
H10, H11	77,720, 73,440		0.721	4.258	3.182	1.064	1.640	2.289
H12, H13, H14	16,380, 10,720, 10,500		1.801	7.221	6.961	1.947	5.285	5.755
H15,H16	14,700, 18,180		1.604	6.430	6.186	1.732	4.683	4.9
H17,H18	26,200, 26,740		1.334	5.350	5.127	1.438	3.865	3.727
H19, H20	77,840, 70,080		1.092	4.377	4.174	1.174	3.116	2.706
H21, H22	152,380, 95,040		0.939	3.764	3.578	1.008	2.633	2.118
H26	2758		1.370	5.481	4.646	1.406	3.283	3.485
H28, H29	535,540, 611,720		0.353	1.412	2.809	0.703	2.384	1.337
**Dynamic Load**								
P2	192,520		0.224	0.894	0.736	0.227	0.386	0.203
P4	23,780		0.360	1.438	1.190	0.366	0.678	0.412
P5	197,190		0.089	0.357	0.708	0.177	0.465	0.165
P6	211,900		0.094	0.378	0.749	0.187	0.496	0.179
P7	17,760		0.111	0.446	0.884	0.221	0.602	0.227
P8	547,680		0.076	0.304	0.603	0.151	0.387	0.130
P9, P10, P11	77,320, 88,240, 36,600		0.192	1.173	0.844	0.293	0.330	0.265
P17, P18	219,500, 263,720		0.128	0.782	0.568	0.195	0.208	0.125
P40, P41	38,360, 63,100		0.278	1.111	1.048	0.297	0.637	0.316
P46	1,002,160		0.178	0.711	0.671	0.190	0.378	0.165

**Table 6:** The correlation coefficient $\varrho_{xy}$ and the regression curve of Equation (16) for parameters A,B.

	Kinematic Load			Dynamic Load		
**Relation**	$\varrho_{\mathrm{xy}}$	**A**	**B**	$\varrho_{\mathrm{xy}}$	**A**	**B**
SWT (5)	−0.91	4.65	−2.36	−0.49	4.08	−1.24
Chu (6)	−0.97	5.08	−3.38	−0.77	2.88	−3.45
Liu ${}^{\left(I\right)}$ (7)	−0.96	6.24	−2.33	−0.47	4.93	−1.07
Liu ${}^{\left(II\right)}$ (8)	−0.92	6.89	−3.44	−0.80	4.63	−4.12
Glinka (9)	−0.62	5.54	−1.97	−0.51	4.45	−1.57
Ellyin (11)	−0.97	5.87	−2.37	−0.79	3.40	−2.46
